# Anogenital HIV RNA in Thai men who have sex with men in Bangkok during acute HIV infection and after randomization to standard vs. intensified antiretroviral regimens

**DOI:** 10.7448/IAS.18.1.19470

**Published:** 2015-05-07

**Authors:** Nittaya Phanuphak, Nipat Teeratakulpisarn, Frits van Griensven, Nitiya Chomchey, Suteeraporn Pinyakorn, James LK Fletcher, Rapee Trichavaroj, Supanit Pattanachaiwit, Nelson Michael, Praphan Phanuphak, Jerome H Kim, Jintanat Ananworanich, on behalf of the RV254/SEARCH 010 Study Group

**Affiliations:** 1 The Thai Red Cross AIDS Research Centre, Bangkok, Thailand; 2 SEARCH, Bangkok, Thailand; 3 HIV-NAT, Bangkok, Thailand; 4 Armed Forces Research Institute of Medical Sciences, Bangkok, Thailand; 5 US Military HIV Research Program, Bethesda, MD, USA; 6 Walter Reed Army Institute of Research Silver Spring, MD, USA; 7 The Henry M. Jackson Foundation for the Advancement of Military Medicine Bethesda, MD, USA

**Keywords:** acute HIV, MSM, anogenital, HIV RNA, antiretroviral therapy, Asia

## Abstract

**Introduction:**

HIV transmission risk is highest during acute HIV infection (AHI). We evaluated HIV RNA in the anogenital compartment in men who have sex with men (MSM) during AHI and compared time to undetectable HIV RNA after three-drug versus five-drug antiretroviral therapy (ART) to understand risk for onward HIV transmission.

**Methods:**

MSM with AHI (*n=*54) had blood, seminal plasma and anal lavage collected for HIV RNA at baseline, days 3 and 7, and weeks 2, 4, 12 and 24. Data were compared between AHI stages: 1 (fourth-generation antigen-antibody combo immunoassay [IA]–, third-generation IA–, *n=*15), 2 (fourth-generation IA+, third-generation IA–, *n=*9) and 3 (fourth-generation IA+, third-generation IA+, western blot–/indeterminate, *n=*30) by randomization to five-drug (tenofovir+emtricitabine+efavirenz+raltegravir+maraviroc, *n=*18) versus three-drug (tenofovir+emtricitabine+efavirenz, *n=*18) regimens.

**Results:**

Mean age was 29 years and mean duration since HIV exposure was 15.4 days. Mean baseline HIV RNA was 5.5 in blood, 3.9 in seminal plasma and 2.6 log_10_ copies/ml in anal lavage (*p*<0.001). Blood and seminal plasma HIV RNA were higher in AHI Stage 3 compared to Stage 1 (*p*<0.01). Median time from ART initiation to HIV RNA <50 copies/ml was 60 days in blood, 15 days in seminal plasma and three days in anal lavage. Compared with the three-drug ART, the five-drug ART had a shorter time to HIV RNA <1500 copies/ml in blood (15 vs. 29 days, *p=*0.005) and <50 copies/ml in seminal plasma (13 vs. 24 days, *p=*0.048).

**Conclusions:**

Among MSM with AHI, HIV RNA was highest in blood, followed by seminal plasma and anal lavage. ART rapidly reduced HIV RNA in all compartments, with regimen intensified by raltegravir and maraviroc showing faster HIV RNA reductions in blood and seminal plasma.

## Introduction

The risk of sexual transmission of HIV is highest during acute HIV infection (AHI) [1]. Phylogenetic modelling of circulating viral strains suggests that the proportion of all new HIV infections acquired from AHI individuals could be as high as 50% [2–5]. An outburst of viral replication and rapid dissemination of the virus typical for AHI, as evidenced by extremely high HIV RNA levels and viral homogeneity with a transmitted/founder phenotype in blood and genital secretions, likely contribute to the higher infectiousness during AHI compared to later stage disease [1,6,7].

The AHI period spans the first month of infection, prior to detectable HIV IgG [8]. In the blood, HIV RNA rises rapidly and peaks at three weeks post-infection, generally to levels that reach or exceed 6 log_10_ copies/ml, before declining to a viral set point 1–2 months later [9]. Viral dynamics in seminal plasma during primary HIV infection (within 6–12 months of infection) appear to mimic that of the blood but with a lower HIV RNA level [10,11]. However, in these studies, only a handful of AHI subjects are included, thus limiting our understanding of HIV infectiousness during this period of greatest risk. Data are even more scarce in men who have sex with men (MSM), who are disproportionately infected with HIV in many settings, including Thailand [12]. Moreover, no studies evaluated anorectal HIV RNA, the port of infection entry for receptive anal intercourse. One study in chronically infected MSM found higher HIV RNA levels in the rectal secretions compared to the blood and postulated that it was from higher target cells for HIV and CD4 depletion in the gut [13]. Whether there is sequestration or compartmentalized HIV replication in the anogenital compartment early after viral entry is unknown.

Antiretroviral therapy (ART) is effective in suppressing viremia in the blood to undetectable levels, but up to 30% of well-suppressed patients on protease inhibitor-containing regimens continue to have detectable genital HIV RNA [14]. One explanation is the inadequate penetration of antiretroviral drugs into this compartment, which can vary across gender, drugs and drug classes [15–18]. Reverse transcriptase (RT) inhibitors generally penetrate well, as do the entry and integrase inhibitors, whereas protease inhibitors are less penetrant [19]. It is unclear what regimen is best at suppressing anogenital HIV RNA, particularly during high viremia in AHI.

Here we have a unique opportunity to evaluate HIV RNA in the anogenital compartment (anal lavage and seminal plasma) in MSM during early AHI, as well as after randomization to three-drug standard ART with RT inhibitors versus five-drug ART of standard ART intensified with integrase and entry inhibitors. Knowledge of viral burden in the anogenital compartments in MSM during AHI and after suppressive ART will be relevant to understanding the HIV pathogenesis and risk for onward transmission [11,13,20].

## Methods

The RV254/SEARCH 010 study prospectively screened and enrolled AHI subjects at the Thai Red Cross Anonymous Clinic in Bangkok, Thailand (clinicaltrials.gov identification number NCT00796146). The study was approved by the institutional review boards (IRBs) of the Chulalongkorn University in Thailand and the Walter Reed Army Institute of Research in the United States. Subjects who elected to start ART were controlled in an accompanying local protocol (clinicaltrials.gov identification number NCT00796263), which was approved by the Chulalongkorn University IRB. All subjects gave informed consent.

### Identification and staging of AHI

Samples were screened and staged for AHI according to published methods [21]. AHI subjects had positive HIV RNA in blood plasma and were categorized into three stages using a staging system called “4thG,” which is based on results from the fourth-generation antigen-antibody combo immunoassay (IA) [8]: Stage 1 (fourth-generation antigen-antibody combo IA negative, third-generation IA negative), Stage 2 (fourth-generation IA positive, third-generation IA negative) and Stage 3 (fourth-generation IA positive, third-generation IA positive, western blot negative/indeterminate).

### ART initiation

Subjects who elected to initiate ART were randomized to either three-drug ART (tenofovir 300 mg once daily+emtricitabine 200 mg once daily+efavirenz 600 mg once daily) versus five-drug ART (standard ART+raltegravir 400 mg twice daily+maraviroc 600 mg twice daily).

### Anogenital specimen collection

Semen was self-collected into a container and 3 ml of viral transport medium (VTM) was added. The total volume of the semen specimen was recorded. The specimen was centrifuged at 600 g for 15 minutes. Seminal plasma was separated from the cell pellet and stored at −80°C.

Anal lavage was obtained, through an anoscope, using 2 ml of sterile normal saline solution, which was aspirated after three rounds of wash over the transformation zone. The anal lavage specimen was centrifuged at 600 g for 10 minutes. The supernatant was separated from the cell pellet and stored at −80°C.

### Laboratory assessments

Blood, semen and anal lavage specimens were collected at days 0, 3, 7, weeks 2, 4, 12 and 24. HIV RNA quantification for all specimens was performed using Amplicor^®^ HIV-1 Monitor Test version 1.5 (Roche Molecular Systems, Inc., Branchburg, NJ, USA). The assay's lower limit of detection (LLOD) was 50 copies/ml. For seminal plasma, HIV RNA levels were adjusted for the dilution in VTM. The dilution factor was not taken into account for the HIV RNA quantification of the anal lavage specimen. All specimens with HIV RNA levels below LLODs were assigned censored values.

Blood collected on day 1 was tested for syphilis using rapid plasma reagin test with *Treponema pallidum* haemagglutination confirmation. Anal lavage specimens collected on day 0 were also tested for human papillomavirus (HPV) using a Roche Linear Array assay (Roche Molecular Diagnostics, CA, USA), which detects 37 anogenital HPV DNA genotypes, including 13 oncogenic high-risk types (16, 18, 31, 33, 35, 39, 45, 51, 52, 56, 58, 59 and 68), for herpes simplex virus (HSV) using a LightMix^®^ Kit HSV-1/2 (Roche Diagnostics, Berlin, Germany), and for gonorrhoea and chlamydia using in-house nucleic acid amplification tests as previously described [22,23].

### Statistical analysis

For this analysis, we included only male AHI subjects who reported having sex with male partners and initiated ART. Baseline characteristics of the participants were summarized by means and standard deviations for continuous variables, whereas numbers and percentages were used for categorical variables. Normality of continuous variables was checked graphically and by Shapiro-Wilk W test. HIV RNA in blood and seminal plasma were normally distributed. HIV RNA in anal lavage appeared to have non-normal distribution, but log_10_ transformation did improve the distribution, compared to the original scale. Here we decided to report HIV RNA in anal lavage by mean and standard deviation to make it consistent with the other two compartments. The relationship between HIV RNA levels in different compartments at baseline was assessed by Pearson's correlation. HIV RNA levels between the 4thG stages and the compartments were compared by t-test. Shapiro-Wilk test was used to assess normality of HIV RNA levels in each group. The t-test was done on the transformed HIV RNA. A non-parametric Mann-Whitney U test was also done. The results from the two methods were consistent. Linear regression model and backward stepwise elimination were used to identify factors associated with HIV RNA levels in each compartment. With regard to backward stepwise elimination, candidate predictors were those with *p*-values less than 0.1 in univariate analysis. A stopping rule of α=0.05 was used; that is, the predictors with a *p*-value greater than the threshold were excluded from the final model. All the assumptions were held. The residuals were normally distributed. Heteroscedasticity was tested using Breusch-Pagan and Cook-Weisberg test. The variance inflation factor was also calculated.

Subgroup analyses were done for randomized patients with 24 weeks of follow up. Survival analyses were conducted to quantify the median time from baseline to HIV RNA below 50 copies/m for all samples. Additionally, time to blood plasma HIV RNA <1500 copies/ml was also determined as a threshold associated with infectivity from a meta-analysis of transmission risk through heterosexual exposure [24]; HIV RNA levels between treatment groups were compared by t-test. All hypotheses testing were 2-sided tests, at a 5% significant level. All statistical analyses were done using Stata/IC 12.1 for windows (Stata Corporation, College Station, TX, USA).

## Results

### At time of AHI

#### Participant characteristics

Between April 2009 and December 2012, 61,513 samples were prospectively screened for AHI, 100 subjects were diagnosed with AHI and 80 enrolled in this study (Table 1). Of these, 73 were MSM, one was a heterosexual man and six were women. Of the 73 MSM, 71 elected to start ART and 54 who provided anogenital samples were included in this analysis. Of the participants, 15 were in 4thG Stage 1, 9 were in 4thG Stage 2, and 30 were in 4thG Stage 3. Their mean (SD) age was 29 (6.5) years old. The mean (SD) duration since time of HIV exposure was 15 (6.4) days. Forty-five MSM (83%) were experiencing acute retroviral syndrome. The mean (SD) CD4 count was 422 (207) cells/mm^3^ and 78% were infected with CRF01_AE clade.

**Table 1:** Baseline characteristics of 54 men who have sex with men with acute HIV infection

	At enrolment (*n=*54)				At 24 weeks after randomization to 3- vs. 5-drug ART (*n=*36)	
	
Baseline characteristics	Total (*n=*54)	4thG1 (*n=*15)	4thG2 (*n=*9)	4thG3 (*n=*30)	3-drug ART (*n=*18)	5-drug ART (*n*=18)
Mean age, years (SD)	29.0 (6.5)	29.3 (8.9)	31.0 (4.7)	28.3 (5.5)	30.9 (7.4)	27.7 (6.9)
4thG stage, *n* (%)						
1 (RNA+/4th gen IA–/3rd gen IA–)	15 (27.8)	–	–	–	6 (33.3)	7 (38.9)
2 (RNA+/4th gen IA+/3rd gen IA–)	9 (16.7)	–	–	–	1 (5.6)	1 (5.6)
3 (RNA+/3rd gen IA+/western blot– or indeterminate)	30 (55.5)	–	–	–	11 (61.1)	10 (55.6)
Mean time since HIV exposure, days (SD)	15.4 (6.4)	11.3 (4.3)	16.4 (4.6)	17.1 (6.9)	16.8 (5.1)	15.2 (7.4)
Acute retroviral syndrome, *n* (%)	45 (83.3)	11 (73.3)	8 (88.9)	26 (86.7)	14 (77.8)	16 (88.9)
CD4 count (cells/mm^3^)	422 (207.1)	477 (237.1)	367 (168.8)	411 (201.8)	408 (229.8)	462 (123.1)
HIV subtype (*n=*48), *n* (%)						
CRF01_AE	42 (77.8)	11 (73.3)	7 (77.8)	24 (80.0)	16 (88.8)	13 (72.2)
B	2 (3.7)	1 (6.7)	1 (11.1)	0 (0)	0 (0)	1 (5.6)
CRF01_AE/B recombinant	3 (5.5)	1 (6.7)	0 (0)	2 (6.7)	1 (5.6)	2 (11.1)
Non-typable	7 (13.0)	2 (13.3)	1 (11.1)	4 (13.3)	1 (5.6)	2 (11.1)
Tropism (*n*=54), *n* (%)						
R5	37 (68.5)	9 (60.0)	9 (100.0)	19 (63.3)	12 (66.7)	12 (66.7)
X4	2 (3.7)	0 (0)	0 (0)	2 (6.7)	1 (5.6)	0 (0)
Non-typable	15 (27.8)	6 (40.0)	0 (0)	9 (30.0)	5 (27.7)	6 (33.3)
Route of HIV acquisition						
Insertive anal sex only	4 (7.4)	3 (20.0)	1 (11.1)	0 (0)	1 (5.6)	2 (11.1)
Receptive anal sex only	30 (55.6)	7 (46.7)	5 (55.6)	18 (60.0)	10 (55.6)	8 (44.4)
Both insertive and receptive sex	12 (22.2)	3 (20.0)	2 (22.2)	7 (23.3)	5 (27.8)	5 (27.8)
Insertive or receptive oral sex	8 (14.8)	2 (13.3)	1 (11.1)	5 (16.7)	2 (11.1)	3 (16.7)
Mean number of sexual partners in the past month, (SD)	3 (2)	3 (4)	2 (1)	2 (2)	3 (3)	2 (1)
Single partner, *n* (%)	20 (37.0)	7 (46.7)	1 (11.1)	12 (40.0)	5 (27.8)	9 (50.0)
Multiple partners, *n* (%)	34 (63.0)	8 (53.3)	8 (88.9)	18 (60.0)	13 (72.2)	9 (50.0)
Alcohol and/or illicit drug use with sex in the past month, *n* (%)	20 (37.0)	6 (40.0)	5 (55.6)	9 (30.0)	7 (38.9)	4 (22.2)
Circumcised, *n* (%)	5 (9.3)	3 (20.0)	1 (11.1)	1 (3.3)	0 (0.0)	3 (16.7)
Sexually transmitted infections						
Syphilis, *n* (%)	5 (9.3)	1 (6.7)	0 (0)	4 (13.3)	4 (22.2)	0 (0)
Anal chlamydia, *n* (%)	2 (3.7)	2 (13.3)	0 (0)	0 (0)	0 (0)	0 (0)
Anal gonorrhoea, *n* (%)	0 (0)	0 (0)	0 (0)	0 (0)	0 (0)	0 (0)
Anal HSV infection, *n* (%)	0 (0)	0 (0)	0 (0)	0 (0)	0 (0)	0 (0)
Anal HPV infection, *n* (%)	20 (37.0)	5 (33.3)	1 (12.5)	14 (46.7)	5 (27.8)	7 (38.9)
HIV RNA						
Blood plasma, log_10_ copies/ml (*N=*54)	5.5 (1.16)	4.5 (0.96)	5.9 (0.64)^a^	5.9 (1.05)^a^	5.5 (1.23)	5.2 (1.27)
*n* (%)<50 copies/ml	0 (0)	0 (0)	0 (0)	0 (0)	0 (0)	0 (0)
Seminal plasma, log_10_ copies/ml (*N=*44)	3.9 (1.25)	3.4 (1.65)	4.0 (0.87)	4.1 (1.04)	4.2 (1.24)	3.8 (1.26)
*n* (%) <50 copies/ml	3 (6.8)	3 (23.1)	0 (0)	0 (0)	0 (0)	1 (5.9)
Anal lavage, log_10_ copies/ml (*N=*52)	2.6 (0.77)	2.7 (1.15)	3.0 (0.48)	2.5 (0.57)	2.6 (0.74)	2.3 (0.49)
*n* (%) <50 copies/ml	11 (21.2)	6 (40.0)	0 (0)	5 (16.7)	4 (22.2)	6 (33.3)

a 4th gen IA, fourth-generation antigen-antibody combo immunoassay; 3rd gen IA, third-generation HIV IgM-sensitive immunoassay.

ART, antiretroviral therapy.

The majority reported receptive anal intercourse as the likely route of HIV acquisition. In the past month, most had had multiple partners and one-third reported alcohol and/or recreational drug use during sex. Few were circumcised and 9% had syphilis. Prevalence rates of anal sexually transmitted infections (STIs) were low (anal chlamydia was found in 4% and none had anal gonorrhoea or HSV infection). However 37% had anal HPV infection and 22% were shown to have one or more high-risk HPV types.

#### HIV RNA levels between 4thG stages

**Figure 1:**
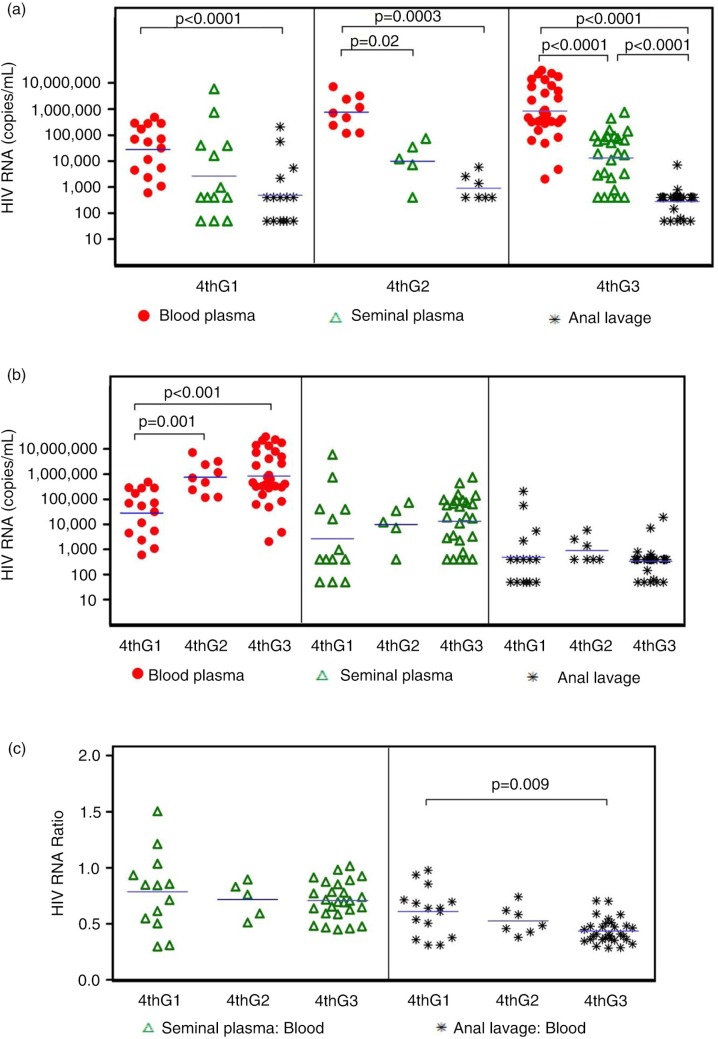
Comparison of HIV RNA levels in blood plasma, seminal plasma and anal lavage by (a) 4thG stage, (b) compartment and (c) genital/blood ratio.

HIV RNA levels were highest in blood plasma, followed by seminal plasma and anal lavage, for all AHI stages (Figure 1a). Mean HIV RNA values were 5.5 log_10_ copies/ml for blood plasma, 3.9 log_10_ copies/ml for seminal plasma and 2.6 log_10_ copies/ml for anal lavage (Table 1). All subjects had detectable blood plasma HIV RNA, whereas 7 and 21% had HIV RNA below 50 copies/ml in the seminal plasma and anal lavage, respectively.

Figure 1b demonstrates the comparison by compartment for HIV RNA levels across 4thG stages. Blood plasma HIV RNA increased with higher 4thG stage (*p=*0.001 comparing 4thG Stage 1 and Stage 2, *p*<0.001 comparing 4thG Stage 1 and Stage 3), whereas levels were similar across AHI stages in the other two compartments. No difference was seen in blood plasma HIV RNA between AHI Stage 2 and Stage 3. When ratios of HIV RNA levels between each anogenital compartment versus blood plasma were evaluated (Figure 1c), we observed a higher anal lavage/blood plasma HIV RNA ratio in 4thG Stage 1 versus Stage 3 (*p=*0.009).

In multivariate analysis, factors associated with higher HIV RNA levels in blood plasma included being in 4thG Stage 2 or Stage 3 or having a CD4 count ≤350 cells/mm^3^. Higher HIV RNA levels in blood plasma were associated with higher HIV RNA levels in seminal plasma (Table 2).

**Table 2:** Univariate and multivariate analyses of factors associated with HIV RNA levels in different compartments, by a simple linear regression

	HIV RNA in plasma (log_10_ copies/ml)				HIV RNA in seminal plasma (log_10_ copies/ml)				HIV RNA in anal lavage (log_10_ copies/ml)			
	
	Univariate		Multivariate		Univariate		Multivariate		Univariate		Multivariate	
	
Factors	Coefficient (95% CI)	*p*	Coefficient (95% CI)	*p*	Coefficient (95% CI)	*p*	Coefficient (95% CI)	*p*	Coefficient (95% CI)	*p*	Coefficient (95% CI)	*p*
Age (years)												
> 25	Ref.				Ref.				Ref.			
≤ 25	−0.2 (−0.9–0.5)	0.57			0.2 (−0.7–1.0)	0.66			0.3 (−0.2–0.4)	0.25		
4thG stage		**<0.0001**		**<0.0001**		0.26				0.39		
1	Ref.		Ref.		Ref.				Ref.			
2	**1.4 (0.6–2.2)**	**0.001**	**1.3 (0.6–1.9)**	**0.001**	0.6 (−0.7**–**1.9)	0.39			0.3 (−0.4–1.0)	0.44		
3	**1.5 (0.9–2.1)**	**<0.001**	**1.5 (0.9–2.0)**	**<0.001**	0.7 (−0.1**–**1.5)	0.10			−0.2 (−0.7–0.3)	0.51		
CD4 count (cells/mm^3^)												
> 350	Ref.		Ref.		Ref.				Ref.			
≤ 350	**1.1 (0.5–1.7)**	**<0.001**	**1.1 (0.6–1.5)**	**<0.001**	**0.8 (0.1–1.6)**	**0.03**			0.4 (−0.1–0.8)	0.10		
HIV RNA in blood plasma	NA	NA			**0.4 (0.1–0.7)**	**0.006**	**0.4 (0.1–0.7)**	**0.006**	0.1 (−0.1–0.3)	0.32		
HIV RNA in seminal plasma	0.5 (0.2–0.8)	0.003			NA	NA			−0.1 (−0.3–0.1)	0.19		
HIV RNA in anal lavage	0.2 (−0.2–0.6)	0.32			−0.3 (−0.8**–**0.2)	0.19			NA	NA		
Number of sexual partners												
Single partner	Ref.				Ref.				Ref.			
Multiple partners	0.1 (−0.6**–**0.8)	0.76			0.1 (−0.7–1.0)	0.73			0.4 (−0.1–0.8)	0.11		
Route of HIV acquisition		0.63				0.56				0.88		
Anal insertive	Ref.				Ref.				Ref.			
Anal receptive	0.8 (−0.5–2.0)	0.23			−0.9 (−2.5–0.6)	0.24			0.1 (−0.9–1.0)	0.86		
Both anal insertive and receptive	0.7 (−0.6–2.1)	0.28			−0.5 (−2.1–1.2)	0.59			−0.1 (−1.2–0.9)	0.80		
Oral insertive and receptive	0.9 (−0.5–2.3)	0.22			−0.9 (−2.6–0.9)	0.32			0.1 (−1.0–1.2)	0.88		
Alcohol or illicit drug use with sex												
No	Ref.				Ref.				Ref.			
Yes	0.2 (−0.5–0.9)	0.55			0.1 (−0.7–0.9)	0.78			0.1 (−0.4–0.5)	0.72		
HPV infection												
No	Ref.				Ref.				Ref.			
Yes	1.1 (0.4–1.8)	0.003			−0.1 (−1.0–0.9)	0.87			0.1 (−0.5–0.7)	0.71		
Circumcised												
No	Ref.				Ref.				Ref.			
Yes	−0.5 (−1.6–0.6)	0.35			0.2 (−1.2–1.5)	0.81			−0.4 (−1.1–0.3)	0.29		
Acute retroviral syndrome												
No	Ref.				Ref.				Ref.			
Yes	0.7 (−0.1–1.6)	0.09			0.8 (−0.3–1.8)	0.14			0.2 (−0.4–0.7)	0.59		

CI, confidence interval.

Bold values are significant at *p*<0.05.

### At 24 weeks after randomization to three- versus five-drug ART

#### Time to undetectable HIV RNA in blood plasma, seminal plasma and anal lavage

**Figure 2:**
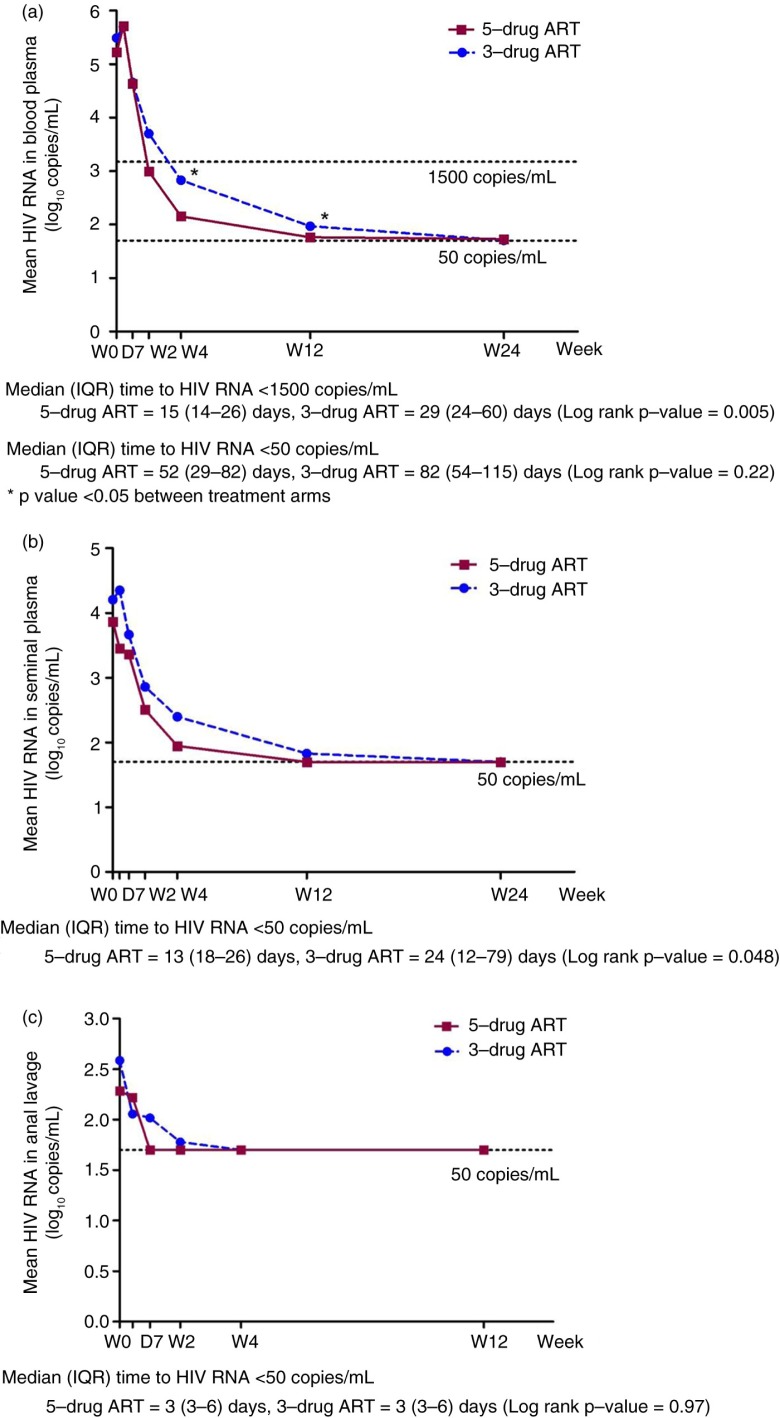
Comparison of HIV RNA decline between five-drug versus three-drug antiretroviral therapy: (a) HIV RNA in blood plasma, (b) HIV RNA in seminal plasma and (c) HIV RNA in anal lavage.

Subgroup analyses were done for 36 randomized subjects who completed 24 weeks of follow-up, 18 per treatment arm (Table 3 and Figure 2). The two groups had similar baseline characteristics (Table 1). ART initiation occurred at a median (interquartile range, IQR) time of 2 (1–4) days after the study enrolment. The median (IQR) time from ART initiation to HIV RNA level below 50 copies/ml was 60 (37–109) days in blood plasma, 15 (10–40) days in seminal plasma and 3 (3–6) days in anal lavage. For blood plasma, the median time to HIV RNA <1500 copies/ml, a threshold associated with infectivity from a meta-analysis of transmission risk through sexual exposure [24], was 24 (14–36) days. As is shown in Figure 2a, the five-drug ART group had a steeper decline in HIV RNA in blood plasma than the three-drug ART; consequently, it took 15 days for the HIV RNA to fall below 1500 copies/ml, compared to 29 days in the three-drug ART group (*p=*0.005). The median time to HIV RNA <50 copies/ml was not significantly different between groups (52 days in the five-drug group vs. 82 days in the three-drug group, *p*=0.22). In seminal plasma (Figure 2b), the time to HIV RNA <50 copies/ml was shorter in the five-drug group (13 days vs. 24 days, *p=*0.048). For anal lavage (Figure 2c), the time to HIV RNA undetectability did not differ between the two groups. By 24 weeks of ART, HIV RNA was undetectable in the seminal plasma and anal lavage of all cases. All except one patient had undetectable HIV RNA in the blood at week 24.

**Table 3:** Median time to HIV RNA decline in blood plasma, seminal plasma and anal lavage in 36 men who have sex with men with acute HIV infection who were randomized to receive three-drug or five-drug ART

	Total (*n=*36)	3-drug ART (*n=*18)	5-drug ART (*n=*18)	*p*
Blood plasma				
Time to HIV RNA <1500 copies/ml, days (IQR)	24 (14–36)	29 (24–60)	15 (14–26)	0.005
Time to HIV RNA <50 copies/ml, days (IQR)	60 (37–109)	82 (54–115)	52 (29–82)	0.22
Seminal plasma				
Time to HIV RNA <50 copies/ml, days (IQR)	15 (10–40)	24 (12–79)	13 (8–26)	0.048
Anal lavage				
Time to HIV RNA <50 copies/ml, days (IQR)	3 (3–6)	3 (3–6)	3 (3–6)	0.966

ART, antiretroviral therapy; IQR, interquartile range.

## Discussions

We demonstrated that HIV RNA levels in MSM were highest in blood, followed by seminal plasma and anal lavage, across all stages of AHI. Treatment with the five-drug regimen, which was intensified by integrase and entry inhibitors, resulted in a faster decline in HIV RNA in blood and seminal plasma than the three-drug regimen, with nucleoside and non-nucleoside RT inhibitors only. Such rapidity in HIV RNA decline may be beneficial in reducing infectiousness in persons with continued engagement in high-risk behaviour.

The higher HIV RNA levels in blood compared to seminal plasma are consistent with previously reported data [1,10,11]. A US study showed that, in 110 males with primary HIV infection (within eight months of HIV infection), the peak HIV RNA levels were 5.3 log_10_ copies/ml in blood versus 4.5 log_10_ copies/ml in seminal plasma, whereas the viral set-points were 4.2 versus 3.5 log_10_ copies/ml in these two compartments, respectively [10]. In that study, only nine men had their samples collected within the first month of infection, the time frame of our sampling. Previous reports have not evaluated HIV RNA in anal lavage in AHI subjects. In our study, seminal plasma HIV RNA was on average 1.3 log_10_ copies/ml higher than in the anal lavage. The lower HIV RNA in anal lavage compared to the seminal plasma is consistent with the lower risk for insertive anal intercourse (11 infections per 10,000 exposures) compared to that of receptive anal intercourse (138 infections per 10,000 exposures) [25]. Compared to a study among chronically infected Thai men, our AHI patients had higher blood (5.5 vs. 4.1 log_10_ copies/ml) and seminal HIV RNA (3.9 vs. 2.5 log_10_ copies/ml) [14]. Aside from HPV, overall our patients had a low rate of anal STIs, which precluded any evaluation of the association between anal STIs and HIV RNA in anal lavage in this study. STIs can lead to immune activation and an increase in HIV replication [26]. Other studies have shown anal chlamydia to be associated with detectable HIV RNA in the anal compartment of MSM prior to, but not after, ART initiation [27,28]. The relevance of HPV infection to HIV replication in the genital compartment is unclear, although limited evidence suggests little to no interaction [29,30].

The higher blood plasma HIV RNA in the later stages of AHI is well documented [9]. Peak viremia occurs around three weeks following infection, which coincides with 4thG Stage 3. Although the HIV RNA was not different across stages for the other two compartments, the higher ratio of anal lavage and blood plasma HIV RNA in 4thG Stage 1 versus Stage 3 could be suggestive of local viral replication occurring at the most likely port of HIV entry in MSM during early AHI. When we performed analyses using the Fiebig staging system [31], the results were similar to data with the 4thG staging (data not shown). The 4thG staging differs from the Fiebig staging in that it distinguishes two groups of Fiebig I individuals by negative (4thG Stage 1) and positive (4thG Stage 2) fourth-generation antigen-antibody IA. These two groups appear to have different proviral and viral burden, which may be relevant to HIV remission and prevention research. The 4thG system also has an advantage over the Fiebig staging system in that it does not require testing with second-generation HIV IgG-sensitive IA, the manufacturing of which is being phased out [8].

ART has been conclusively shown in the HPTN 052 study to reduce HIV transmission by 96% in heterosexual couples [32]. Diagnosis and treatment of AHI could reduce the number of new HIV cases [33,34]. In our study, ART rapidly suppressed HIV RNA in all compartments. It took a median of 3 and 15 days to become undetectable in the anal lavage and the seminal plasma, respectively, whereas this took 60 days in the blood. Without treatment, the seminal plasma HIV RNA in a US male cohort was above 3.8 log_10_ copies/ml for two months or more following primary HIV infection [10]. Scientific evidence strongly supports the HIV preventive benefits of ART in heterosexuals; it is therefore possible that the same effects could be observed for the MSM population. In Thailand, a large test-and-treat demonstration project is being conducted among MSM to close the knowledge gap on this critical scientific and public health issue (Clinicaltrials.gov NCT01869595).

Although the HIV RNA threshold for diminished sexual transmission risk of HIV is not known precisely, the Rakai study [6] showed no transmission when blood plasma HIV RNA was below 1500 copies/ml among heterosexuals. It is well known that the risk of per-contact transmission for anal intercourse is higher than that for vaginal intercourse. However, knowledge on the association between HIV RNA levels and transmission risk among MSM is sorely lacking, and data from a heterosexual population may not be representative of such an association in the setting of MSM. Here, we observed that the five-drug ART had a shorter time to HIV RNA below 1500 copies/ml in blood and below 50 copies/ml in seminal plasma compared to three-drug ART. This observation has potential practical and policy implications for the use of the five-drug regimen, particularly when there is an imminent risk of further transmission, such as during acute seroconversion. Previous studies have demonstrated good concentrations of nucleoside analogues [16,35] and efavirenz [36], used in both treatment groups, in male genital secretions. The addition of maraviroc and raltegravir, which appear to have good penetration in the genital compartment, may explain the faster decline of HIV RNA in seminal plasma with the five-drug regimen. The concentration of raltegravir was 1.42-fold (range 0.52–6.66) higher in semen than in blood in one study [17], whereas active maraviroc concentrations were more than twofold higher in seminal plasma than in blood plasma [37] and 7.5- to 26-fold higher in rectal tissue than in blood [37].

Our study has some limitations. We quantified HIV RNA in the anal compartment using anal lavage samples, which are subject to variable dilutions. Direct collection of secretions using swabs or absorbent wicks, which was not done in this study, may increase the rate of HIV detection in genital fluids. There could be other STIs, such as cytomegalovirus, affecting HIV RNA levels that were not tested [26]. As almost all MSM in the study elected to initiate ART, we do not have comparative data from untreated subjects. The sample size is small. However, the inclusion of MSM during the earliest stages of HIV infection, the frequent sampling of blood, semen and anal specimens that was done on the same day, and the randomization to standard and intensified ART are major strengths of our study.

## Conclusions

In summary, we demonstrated higher HIV RNA levels in blood, followed by seminal plasma and anal lavage, in MSM with AHI. HIV RNA was highest in later stages of AHI in blood and semen. At 24 weeks of ART, HIV RNA was below 50 copies/ml in the anogenital compartment in all patients. The regimen used in our study was intensified with maraviroc and raltegravir and had a shorter time to viral undetectability in blood and seminal plasma, which raises the possible benefit of using these drug classes as part of either a standard or an intensified regimen during high viremia in AHI to reduce risk for onward HIV transmission. Cost-effectiveness studies may help to guide policy decisions on the use of intensified regimen in the public health setting during periods of high transmission risk.
